# Extremity Radioactive Iodine Uptake on Post-therapeutic Whole Body Scan in Patients with Differentiated Thyroid Cancer

**Published:** 2015

**Authors:** Hiroshi Wakabayashi, Junichi Taki, Anri Inaki, Ayane Toratani, Daiki Kayano, Seigo Kinuya

**Affiliations:** Department of Nuclear Medicine, Kanazawa University, Kanazawa, Ishikawa, Japan

**Keywords:** Lower Extremity, Physiological Uptake, Radioactive Iodine, Thyroid Cancer, Whole Body Scan

## Abstract

**Objective(s):**

We investigated a frequency of lower extremity uptake on the radioactive iodine (RAI) whole body scan (WBS) after RAI treatment in patients with differentiated thyroid cancer, in order to retrospectively examine whether or not the frequency was pathological.

**Methods:**

This retrospective study included 170 patients with thyroid cancer, undergoing RAI treatment. Overall, 99(58%) and 71(42%) patients received single and multiple RAI treatments, respectively. Post-therapeutic WBS was acquired after 3 days of RAI administration. For patients with multiple RAI treatments, the WBS of their last RAI treatment was evaluated. Lower extremity uptake on post-therapeutic WBS was classified into 3 categories: bilateral femoral uptake (type A), bilateral femoral and tibia uptake (type B), and uptake in bilateral upper and lower extremities (type C). Then, the patients with RAI uptake in the lower extremities on WBS were analyzed with clinical parameters.

**Results:**

Overall, 99 patients (58%) had the extremity uptake on their posttherapeutic RAI WBS. As the results indicated, 42, 53, and 4 patients had type A, type B, and type C uptakes, respectively. Lower extremity uptake was significantly associated with younger age, not only in subjects with multiple RAI treatments but also in all the patients (P<0.05). Accumulation in patients with multiple RAI treatments was more frequent than patients with single RAI treatment (P<0.05). Lower extremity uptake was not associated with counts of the white blood cell count, hemoglobin level, platelet count, estimated glomerular filtration rate, effective half-time of RAI, serum TSH level, and anti-Tg concentration.

**Conclusion:**

About half of the patients had lower extremity uptake on the posttherapeutic RAI WBS, especially younger patients and those with multiple courses of RAI treatment. Bilateral lower extremity’s RAI uptake on the posttherapeutic WBS should be considered as physiological RAI distribution in bone marrow.

## Introduction

Postoperative radioactive iodine (RAI) ablation is known as an effective method to destroy the remaining thyroid cells and reduce the recurrence of papillary and follicular thyroid cancers, with little damage to other normal tissues. Post-therapeutic RAI whole body scan (WBS) can identify the location of tumors and provide evidence of metastatic radioiodine avidity. Therefore, accurate reading of post-therapeutic WBS is important (1).

Non-metastatic radioiodine-avidity uptakes in breasts, inflammatory lung disease, cholecystitis, and sebaceous cysts are reported as metastasis mimicking uptakes in patients with thyroid cancer (2-5). However, as for the bone marrow uptake of lower extremities, only one such case has been reported by Bohnen and colleagues (6). Therefore, we investigated a frequency of lower extremity uptake on the post-therapeutic RAI WBS to retrospectively examine whether or not the frequency was pathological.

## Methods

We assessed a frequency of lower extremity RAI uptake in 170 patients with differentiated thyroid cancer (57 males and 113 females), who had received RAI therapy between January 2006 and December 2010. Patients had histologically confirmed differentiated thyroid cancer. Overall, 1, 4, 11, and 154 cases of oxyphilic, papillary and follicular, follicular, and papillary carcinomas were reported, respectively.

Patient characteristics are presented in Table 1. Four weeks after L-thyroxine withdrawal, RAI treatment was performed with 3.7 GBq in 142 patients, with 5.3 GBq in 19 patients, and with 7.4 GBq in 9 patients. For 71 patients (42%) with multiple RAI treatments, WBS of their last RAI treatment was assessed.

**Table 1:** Patients’ baseline characteristics (n=170)

Age (years)	
Mean±SD	54 ± 15
Range	15–79
Administered RAI dose (GBq)	
Mean ± SD	4.1 ± 0.9
Range	3.7–7.4
Accumulated RAI dose (GBq)	
Mean ± SD	7.0 ± 6.8
Range	3.7–51.8
Frequency of RAI treatment	Number of patients
1	99
2	55
3	11
4	2
5	1
6	1
8	1

RAI: radioactive iodine

Serum thyroid-stimulating hormone (TSH), thyroglobulin (Tg), and anti-thyroglobulin antibody (anti-Tg) concentrations were measured, using chemiluminescent immunoassay systems. All but 2 patients had a TSH concentration of 30 μU/ml or more before RAI administration. Since 2 patients had a serum TSH concentration of less than 30 μU/ml (28 and 29 μU/ml, respectively), RAI was administered after the informed consents were obtained.

Blood cell count and serum creatinine level were also evaluated before RAI administration. For estimating the glomerular filtration rate (GFR), a Japanese equation for estimated GFR (eGFR) was used (7):

eGFR = A×194 × serum creatinine^−1.094^ × age^−0.287^

where eGFR is in mL/min/1.73 m^2^, age is in years, serum creatinine is in mg/100 ml, body weight is in kg, and A is 1 for men and 0.739 for women.

Effective half-time of RAI was calculated in 157 patients. For the other 13 patients, we did not have enough data to calculate the effective half-time of RAI.

^99m^Tc-methylene diphosphonate (MDP) and ^201^Tl WBS were routinely performed within 5 days before RAI administration between January 2006 and September 2008. Seventy-three patients (43%) underwent both examinations, and all patients received oral and written information on the routine tests.

### The whole body scan images

RAI images with therapeutic radioactive dose were acquired 3 days after RAI administration, using a dual-head gamma camera, equipped with high-energy parallel-hole collimators and a 1-inch (E.cam Signature, Siemens Medical Solutions, used from January 2006 to April 2007) or a 3/8 inch (SymbiaT6, Siemens Medical Solutions, used from May 2007 to December 2010) NaI crystal. In the WBS, both anterior and posterior images were acquired at a speed of 15 cm/min, using a 256×256 matrix and a 364 keV photopeak with a 15% window.

### Lower extremity uptake

**Figure 1:**
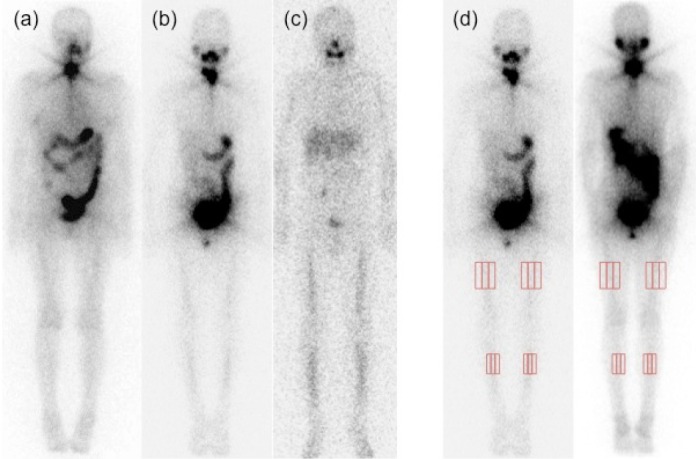
A 64-year-old female patient with type A uptake in the initial RAI therapy (3.7 GBq) (**a**). A 41-year-old female patient with type B uptake in the initial RAI therapy (3.7 GBq) (**b**). A 50-year-old male patient with type C uptake in the second RAI therapy (5.5 GBq) (**c**). Manual ROIs are delineated on images. A rectangular ROI was set manually on bilateral femur and tibia in both anterior and posterior views. As the background, a same-sized ROI was set on both sides (**d**)

Lower extremity uptake was visually evaluated by two experienced nuclear medicine physicians at our institution. They were blinded to patients’ clinical information including their medical history, serum Tg concentration, and prior radiologic test results. For the purpose of analysis, the patterns of bilateral lower extremity uptake were visually classified into 3 categories (Figure 1):

Type A: bilateral femoral uptake,

Type B: bilateral femoral and tibia uptake, and

Type C: uptake in bilateral upper and lower extre-mities.

Semi-quantitative analysis confirmed the lower extremity uptake, as shown in Figure 1. A rectangular region of interest (ROI) was set manually on the middle third portion of bilateral femur and tibia in both anterior and posterior views. As the background, a same-sized ROI was set on both sides. The mean background count density (average count/pixel) of both sides was calculated. Then, the uptake ratio was calculated by dividing the count density of the femur or tibia by that of the background.

If the mean uptake ratio of both anterior and posterior views was more than 1.4, the uptake was reported as significant. The accuracy of visual and semi-quantitative methods was 96% in bilateral femoral uptake and 94% in bilateral tibia uptake.

### Statistical analysis

For statistical analysis, we used a statistical software package (JMP® SAS Institute Inc., Cary, NC, USA). Wilcoxon rank-sum non-parametric test was used to evaluate the median difference. Chi-square test was applied to determine whether there is a significant difference between the expected and observed frequencies in two categories. *P*<0.05 was considered statistically significant.

For analysis, serum Tg concentration less than 0.5 ng/ml was assumed to be 0.5 ng/ml. Similarly, anti-Tg level less than 10 U/mL was interpreted as 10 U/ml.

## Results

Ninety-nine patients (58%) had lower extremity uptake on their post-therapeutic RAI WBS. Overall, 53, 42, and 4 patients had type A, type B, and type C uptakes, respectively.

As shown in Table 2, lower extremity uptake on the images was associated with younger age in all patients (*P*=0.0013). When analysis was restricted to patients with single RAI treatment, lower extremity uptake was mainly reported in younger patients (*P*=0.064), as shown in Table 3. When the analysis was restricted to patients with multiple RAI treatments, younger patients had significant lower extremity uptakes (*P*=0.0013), as shown in Table 4.

**Table 2:** Clinical parameters in all patients and comparison between patients with and without lower extremity uptake

Variables	Patients with bone marrow uptake in lower extremity (n=99)	Patients without bone marrow uptake in lower extremity (n=71)	*P*-value
Age (years)	51 (23-79)	57 (15-78)	0.0013
TSH value (μU/ml)	100 (31-336)	110 (28-317)	0.52
Tg value (ng/ml)	18 (0.5-37642)	61 (0.5-92750)	0.13
anti-Tg value (U/ml)	11 (10-3000)	10 (10-3000)	0.58
Accumulated RAI dose (GBq)	7.4 (3.7-51.8)	3.7 (3.7-22.2)	0.0006
eGFR (ml/min./1.73m^2^)	63 (27-105)	64 (25-126)	0.68
Effective half-time (hr)	11 (6-22)	12 (6-154)	0.064
WBC (×10^3^/μl)	5.1 (2.2-9.4)	5.4 (2.9-10.5)	0.14
Plt (×10^3^/μl)	247 (81-465)	274 (43-423)	0.070
Hb (g/dl)	14 (9-17)	13 (9-18)	0.16

Values are expressed as median and range.

TSH: thyroid-stimulating hormone; Tg: thyroglobulin; anti-Tg: anti-thyroglobulin antibody; eGFR: estimated glomerular filtration rate; min.: minute; WBC: white blood cell; Plts: platelets; Hb: hemoglobin.

**Table 3:** Clinical parameters in patients with single RAI treatment and comparison between patients with and without lower extremity uptake

Variables	Patients with bone marrow uptake in lower extremity (n=44)	Patients without bone marrow uptake in lower extremity (n=55)	*P*-value
Age (years)	49 (23-79)	60 (15-77)	0.064
TSH value (μU/ml)	100 (34-261)	114 (28-317)	0.22
Tg value (ng/ml)	8.7 (0.5-2576)	53 (0.5-3682)	0.029
anti-Tg value (U/ml)	13 (10-3000)	10 (10-3000)	0.25
eGFR (ml/min./1.73m^2^)	56 (30-96)	63 (27-126)	0.55
Effective half-time (hr)	13 (7-19)	13 (8-56)	0.19
WBC (×10^3^/μl)	5.3 (3.7-8.0)	5.7 (2.9-10.5)	0.33
Plt (×10^3^/μl)	259 (145-465)	275 (43-423)	0.54
Hb (g/dl)	14 (9-17)	14 (9-18)	0.81

Abbreviations are the same as Table 2.

**Table 4:** Clinical parameters in patients with multiple RAI treatments and comparison between patients with and without lower extremity uptake

Variables	Patients with bone marrow uptake in lower extremity (n=55)	Patients without bone marrow uptake in lower extremity (n=16)	*P*-value
Age (years)	53 (24-79)	67 (29-78)	0.0013
TSH value (μU/ml)	103 (31-336)	76 (31-206)	0.058
Tg value (ng/ml)	28 (0.5-37462)	90 (0.5-92750)	0.49
anti-Tg value (U/ml)	10 (10-577)	10 (10-1518)	0.76
Accumulated RAI dose (GBq)	7.4 (5.5-51.8)	7.4 (5.5-22.2)	0.75
eGFR (ml/min./1.73m^2^)	67 (25-105)	76 (35-102)	0.25
Effective half-time (hr)	11 (6-22)	10 (6-154)	0.32
WBC (×10^3^/μl)	4.9 (2.2-9.4)	4.6 (3.3-8.1)	0.85
Plt (×10^3^/μl)	248 (81-341)	266 (195-341)	0.15
Hb (g/dl)	14 (9-17)	13 (11-16)	0.12

Abbreviations are the same as Table 2.

In the analysis of patients with single RAI treatment, lower serum Tg concentration was associated with lower extremity uptake (*P*=0.029). However, lower serum Tg concentration was not statistically associated with lower extremity uptake in patients.

Thirty-three patients (19%) had metastatic radioiodine-avid uptake on their post-therapeutic WBS in the extracervical areas, which was not statistically associated with lower extremity uptake, according to Chi-square test results.

Lower extremity uptake was not associated with white blood cell count, hemoglobin level, platelet count, eGFR, effective half-time of RAI, serum TSH level, and anti-Tg concentration.

As shown in Table 5, no statistical differences were observed between patients with type A and type B uptakes regarding the studied variables with the exception of accumulated RAI doses (dose was higher in patients with type B uptake, *P*=0.0004) and effective half-time of RAI (half-time was longer in patients with type B uptake, *P*=0.036).

**Table 5:** Comparison of clinical parameters in patients with type A and type B uptakes

Variables	Type A (n=53)	Type B (n=42)	*P*-value
Age (years)	53 (23-79)	50 (24-76)	0.42
TSH value (μU/ml)	103 (34-255)	94 (31-336)	0.39
Tg value (ng/ml)	18 (0.5-37642)	14 (0.5-20770)	0.36
Anti-Tg value (U/ml)	13 (10-3000)	10 (10-577)	0.16
Accumulated RAI dose (GBq)	3.7 (3.7-51.8)	7.4 (3.7-40.7)	0.0004
eGFR (ml/min./1.73m^2^)	59 (26-96)	69 (25-105)	0.051
Effective half-time (hr)	12 (6-22)	10 (6-18)	0.036
WBC (×10^3^/μl)	5.0 (3.3-9.4)	5.1 (3.1-9.1)	0.52
Plt (×10^3^/μl)	247 (145-465)	246 (143-411)	0.86
Hb (g/dl)	14 (9-17)	14 (9-17)	0.071

Abbreviations are the same as Table 2.

Seventy-one patients (42%) underwent multiple RAI treatments and 55 cases (77%) had lower extremity uptake on the last therapeutic RAI WBS. The evaluation of former therapeutic data of patients with multiple RAI treatments is shown in Table 6. In WBS review of patients with previous RAI treatments, 17 out of 55 patients (31%) did not have lower extremity uptake on the images. The other 33 patients (60%) had lower extremity uptake in previous WBS. Since 5 out of 55 patients (9%) had undergone initial RAI treatment over a decade ago, we were not able to confirm their previous post-therapeutic images. Lower extremity uptake was observed in 55 of 71 patients (78%) with multiple courses of treatment, compared to 44 of 99 patients (44%) with single RAI treatments (*P*<0.0001).

**Table 6:** Characteristics of patients with multiple RAI treatments

Number	Sex	Evaluation of WBS images		Accumulated dose (GBq)	Frequency of RAI treatments
		Previous treatments	The last treatment		
1	M	*	C	9.3	2
2	M	No uptake	B	7.4	2
3	F	A	A	7.4	2
4	F	No uptake	No uptake	7.4	2
5	F	*	B	7.4	2
6	M	No uptake	B	11.1	2
7	F	No uptake	No uptake	7.4	2
8	M	B	B	7.4	2
9	F	A	B	7.4	2
10	M	A	B	11.1	2
11	F	B	B	7.4	2
12	F	No uptake	No uptake	7.4	2
13	F	No uptake	No uptake	11.1	2
14	F	No uptake	No uptake	11.1	2
15	M	No uptake	No uptake	11.1	2
16	F	No uptake	B	5.5	2
17	F	No uptake	B	7.4	2
18	F	No uptake	No uptake	7.4	2
19	F	No uptake	B	7.4	2
20	M	No uptake	No uptake	7.4	2
21	F	A	B	7.4	2
22	F	No uptake	No uptake	7.4	2
23	F	A	B	7.4	2
24	F	B	B	7.4	2
25	M	No uptake	No uptake	7.4	2
26	F	No uptake	B	11.1	2
27	M	No uptake	B	7.4	2
28	F	A	A	7.4	2
29	M	A	C	7.4	2
30	M	A	A	11.1	2
31	F	A	C	7.4	2
32	M	A	B	7.4	2
33	F	A	A	7.4	2
34	M	No uptake	A	7.4	2
35	F	A	A	7.4	2
36	F	No uptake	A	7.4	2
37	F	No uptake	B	7.4	2
38	M	A	B	7.4	2
39	F	No uptake	A	7.4	2
40	M	No uptake	A	7.4	2
41	M	No uptake	A	7.4	2
42	M	A	A	7.4	2
43	M	A	B	7.4	2
44	F	*	A	7.4	2
45	M	A	B	7.4	2
46	F	*	B	7.4	2
47	F	*	A	7.4	2
48	M	A	B	14.8	2
49	F	No uptake	B	7.4	2
50	F	No uptake	A	9.3	2
51	M	A	B	7.4	2
52	F	A	A	7.4	2
53	F	*	No uptake	5.5	2
54	F	No uptake	No uptake	7.4	2
55	F	No uptake	A	7.4	2
56	M	A	B	18.5	3
57	M	B	B	11.1	3
58	F	No uptake	No uptake	11.1	3
59	F	*	No uptake	11.5	3
60	M	No uptake	No uptake	16.7	3
61	F	A	B	16.7	3
62	F	No uptake	No uptake	22.2	3
63	M	A	A	11.1	3
64	M	A	C	11.1	3
65	M	A	B	18.5	3
66	M	No uptake	A	22.2	3
67	F	A	B	24.1	4
68	M	B	B	40.7	4
69	F	A	B	40.7	5
70	M	A	B	40.7	6
71	F	A	A	51.8	8

RAI: radioactive iodine; M: male; F: female

* No data

**Figure 2:**
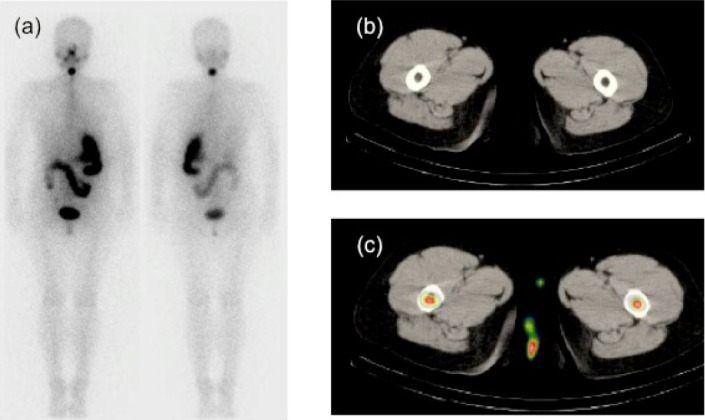
A -41year-old female patient with type B uptake in the initial RAI therapy (3.7 GBq) (**a**). Axial CT (**b**) and fused SPECT/CT (**c**) images show an uptake, localized in the bilateral bone marrow of the femur.

Fused single-photon emission tomography results, along with computed tomography images, suggested bone marrow RAI uptake, as shown in Figure 2. Neither ^99m^Tc-MDP nor ^201^Tl WBS images showed abnormal lower extremity uptake, occurring outside the physiological accumulation.

## Discussion

Post-therapeutic RAI WBS showed bilateral lower extremity uptake in more than half of the patients. Although we suggested three types of lower extremity uptake, all extremity accumulations were fairly infrequent.

Although the process of lower extremity RAI uptake is not clear, distinguishing this accumulation from bone marrow metastasis is of utmost importance. Generally, diffuse bone marrow metastases of differentiated thyroid cancer are uncommon. Anner et al. (8) evaluated bone marrow aspiration in 2,877 patients with solid tumors to determine the presence of clinically unsuspected metastases and found involvement in only 1 out of 33 patients (3%), with known thyroid cancer. Rufini et al. (9) reported rare cases of disseminated bone marrow metastases of insular thyroid carcinoma, detected by RAI WBS. However, in our study, no clinical data suggested the emergence of diffuse bone marrow metastasis. Serum Tg level, measured as part of a routine follow-up (in a hypothyroid state) on the day of RAI administration, was not higher in patients with RAI uptake in lower extremities, compared to those without RAI uptake. Since half of our patients (58%) had bilateral uptake in lower extremities, but not the central bone, it is believed that this type of uptake is not associated with diffuse bone marrow metastasis. Also, 2 to 6 years of follow-up did not show any clinical evidence of metastasis.

According to the published reports, only one case has been reported as normal physiological RAI bone marrow uptake (6). They indicated the possibility of relations between the RAI lower extremity bone marrow uptake and a sporting activity. Although we did not have enough information about the patients’ physical activities, our study did not include professional athletes.

Since younger age was associated with lower extremity uptake, we presumed that existence of red bone marrow had a possible relation to the RAI uptake. Bone marrow is being increasingly converted to yellow marrow with aging. The red marrow, consisting mainly of hematopoietic tissues, is found in the hollow interior of the proximal one-third portion of long bones like extremities (10-12). However, Kim et al. (13) demonstrated that red bone marrow, extending to the proximal one-third portion of the femur, was observed in 147 out of 354 patients (42%) on ^111^In-leukocyte scan images (among different age adult). In their study, red marrow in the entire femur was observed in 42 patients (12%). Lower extremity uptake may be partially related to red marrow. However, we could not realize why central bone marrow did not show any iodine uptake. To the best of our knowledge, no study has yet reported the specific expression of sodium/iodine symporter in bone marrow after RAI treatment.

If the delineation of bone marrow is one of the physiological uptakes, it should decrease with time as do other physiological accumulations in salivary glands, liver, oral cavity, and nose. Hung et al. (14) reported the differences of malignant lesion detection rate on the first (3-4 days after treatment), second (5-6 days after treatment), and third (10-11 days after treatment) WBSs. In their study, 1 out of 2 representative cases had lower extremity uptakes. It is noteworthy that the uptake gradually decreased on triple-phase images. This finding supports the notion that lower extremity uptake is a physiological accumulation. In addition, the frequency and distribution of lower extremity uptake might be affected by the timing of WBS.

## Conclusion

Although the process of lower extremity RAI uptake is not clear, lower extremity RAI uptake was observed on the post-therapeutic WBS in more than half of the patients. Lower extremity RAI uptake tended to be related to younger age and multiple RAI treatments. In clinical practice, lower extremity RAI uptake should not be misinterpreted as metastasis and must reflect normal physiological variants.

### Conflicts of interest

There are no conflicts of interest.
